# Photobiomodulation Therapy at 808 nm Does Not Improve Biceps Brachii Performance to Exhaustion and Delayed-Onset Muscle Soreness in Young Adult Women: A Randomized, Controlled, Crossover Trial

**DOI:** 10.3389/fphys.2021.664582

**Published:** 2021-06-10

**Authors:** Ricardo Henrique Esquivel Azuma, Jeanne Karlette Merlo, Jeferson Lucas Jacinto, Jayne Maria Borim, Rubens Alexandre da Silva, Francis Lopes Pacagnelli, Joao Pedro Nunes, Alex Silva Ribeiro, Andreo Fernando Aguiar

**Affiliations:** ^1^Center for Research in Health Sciences, University of Northern Paraná, Londrina, Brazil; ^2^Département des Sciences de la Santé, Programme de Physiothérapie de L’université McGill Offert em Extension à L’Université du Québec à Chicoutimi, Saguenay, QC, Canada; ^3^Faculty of Health Sciences, Physiotherapy Course, UNOESTE, Presidente Prudente, Brazil; ^4^Metabolism, Nutrition, and Exercise Laboratory, Physical Education and Sport Center, Londrina State University, Londrina, Brazil

**Keywords:** exercise, fatigue, low-level light therapy, pain, physical functional performance, resistance training

## Abstract

**Objective:**

This study aims to investigate the effects of laser photobiomodulation (PBM) at 808 nm on biceps brachii performance to exhaustion, rating of perceived exertion (RPE), and delayed onset muscle soreness (DOMS) in untrained young women.

**Methods:**

Thirteen young women (20.1 ± 2.9 years) participated in a crossover study in which they received, in a counterbalanced manner, active and placebo laser PBM on two occasions (T1 and T2), separated by a 7-day washout period. During T1 and T2, participants received active (100 mW output power, irradiance of 35.7 W cm^–2^, and total energy of 28 J/arm) or placebo laser irradiation on the biceps brachii muscle at 20 min before the repetitions-to-failure test [six sets at 60% of one-repetition maximum (1RM) until failure] for elbow flexion exercise. The number of repetitions performed and RPE over the six sets, as well as DOMS from basal up to 72 h after the repetitions-to-failure test, were recorded.

**Results:**

There was a significant (time, *p* < 0.05) reduction in the number of repetitions performed and an increase in RPE over six sets, with no statistical differences between placebo and active laser conditions (treatment × time, *p* > 0.05). DOMS increased at 24 h postexercise and progressively returned to baseline after 72 h in both conditions (time, *p* < 0.05; treatment × time, *p* > 0.05).

**Conclusion:**

Our results indicate that acute laser PBM at 808 nm does not improve biceps brachii performance to exhaustion, RPE, and DOMS in untrained women.

## Introduction

Ergogenic aids are commonly used by athletes and recreational practitioners in order to improve exercise performance and body composition (Silver, 2001; Goston and Correia, 2010; Senekal et al., 2019). An ergogenic aid is defined as any mechanical, psychological, physiological, pharmacological, or nutritional treatment that enhances energy production and utilization (Williams, 1992) and thus improves exercise performance and recovery (Kerksick et al., 2018). In this context, photobiomodulation (PBM) therapy, also known as low-level laser (light) therapy (LLLT), has emerged as an important non-pharmacological strategy for improving performance (Leal Junior et al., 2008, 2009, 2010; de Almeida et al., 2012; Toma et al., 2018) and recovery (Leal Junior et al., 2010) from exercise in young adults. The theoretical basis underlying the ergogenic effects of PBM on muscle tissue is related to several mechanisms, including (i) increased production of adenosine triphosphate (ATP) via modulation of mitochondrial activity, (ii) stimulation of defenses against oxidative stress, (iii) improvement of regenerative capacity by stimulation of satellite cells, and (iv) possible increased muscle fiber excitability (for more details, see the reviews, de Freitas and Hamblin, 2016; Ferraresi et al., 2016; Hamblin, 2017).

Despite the proposed beneficial mechanisms, few studies to date have investigated the ergogenic effects of PBM on biceps brachii performance and recovery in young adult subjects. While some studies reported an increase in peak force (de Almeida et al., 2012), attenuation in creatine kinase (CK) levels both immediately (Leal Junior et al., 2010) and 72 h after exercise (Felismino et al., 2014), and an increase in the number of repetitions performed at 75% of the maximal voluntary contraction (MVC) (Leal Junior et al., 2008, 2009, 2010), others did not found any beneficial effects on markers of muscle performance (Higashi et al., 2013; Orssatto et al., 2020), fatigue (Leal Junior et al., 2008, 2009; Higashi et al., 2013; Felismino et al., 2014), and recovery [i.e., delayed onset muscle soreness (DOMS) and recovery of strength performance] (Craig et al., 1996, 1999; Felismino et al., 2014). Therefore, conflicting results have been published concerning the effects of PBM.

Such discrepancies might be explained by the methodological differences among the studies, including sex, training status, and laser parameters. For instance, six of these studies included men only (Craig et al., 1996; Leal Junior et al., 2008, 2009, 2010; Felismino et al., 2014), while one included a mix of men and women (Craig et al., 1999), and another included women only (Higashi et al., 2013). Moreover, some studies recruited untrained healthy participants (Craig et al., 1996, 1999; de Almeida et al., 2012; Higashi et al., 2013), while others used volleyball players (Leal Junior et al., 2008, 2009, 2010) or physically active individuals (Felismino et al., 2014). Another point to be considered is that different wavelengths (i.e., 660–950 nm), number of points (i.e., 2–8 points), and total energy (i.e., 4–56 J) were used for the irradiation of biceps brachii in the above-mentioned studies, precluding to establish a consensus on better parameters of laser PBM for inducing ergogenic effects on muscle tissue.

It is also important to note that most studies that found positive results of laser PBM on biceps brachii performance, and recovery parameters included only male participants and used only one set of repetitions until failure (Leal Junior et al., 2008, 2009, 2010) or a single voluntary isometric contraction for 60 s (de Almeida et al., 2012). Considering that multiple sets are frequently applied in a practical context due its greater effect in promoting muscle strength and hypertrophy (ACSM, 2009), it is important to verify the effects of laser in multiple sets exercises. A recent study (Orssatto et al., 2020) found no beneficial effect of PBM on maximum number of repetitions during a resistance exercise session consisting of six sets of repetitions to failure for the standing calf raise exercise in well-trained men and women. In addition, the only study involving exclusively young adult women (Higashi et al., 2013) reported no significant differences between active and placebo laser conditions on electromyography (EMG) fatigue, blood lactate levels, and number of repetitions performed during a 60-s fatigue protocol involving the elbow flexion-extension exercise. The authors did not discuss the influence of sex on the negative findings, but previous studies reported less DOMS after eccentric resistance exercises (Dannecker et al., 2003) and greater resistance to fatigue in women than men during dynamic (Salvador et al., 2005) and isometric (Avin et al., 2010) contractions to failure in the elbow flexion exercise. These findings raise the possibility that a small effect of PBM on biceps brachii muscle performance may be masked by a lower pain intensity and greater muscular endurance in women, particularly under exhaustion conditions (e.g., multiple sets of repetitions to failure). Therefore, further studies are warranted to confirm whether the laser PBM is effective in improving biceps brachii performance to exhaustion and DOMS in young adult women.

The purpose of this study was to investigate the effects of laser PBM at 808 nm on biceps brachii performance to exhaustion and DOMS in untrained young adult women. Based on previous findings in men, we hypothesized that laser PBM would increase the number of repetitions performed, reduce the rating of perceived exertion (RPE), and attenuate DOMS when compared to a placebo laser.

## Materials and Methods

### Study Design

**FIGURE 1:**
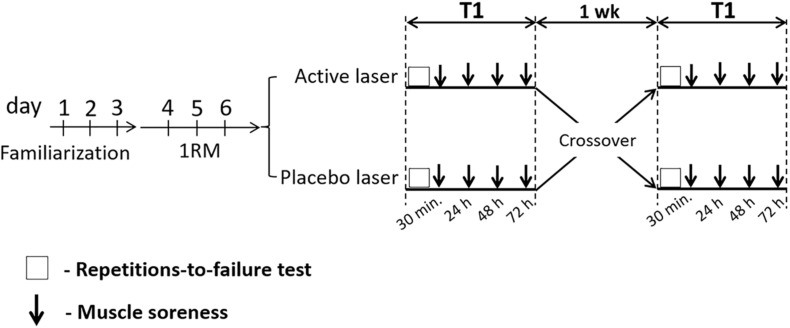
Experimental design.

A crossover, double-blind, randomized, and placebo-controlled study was performed to examine the effects of laser PBM (λ, 808 nm) therapy on biceps brachii performance, RPE, and DOMS in untrained young women. A schematic representation of the experimental design is shown in Figure 1. All participants performed three familiarization sessions for bilateral preacher curl exercise (three sets of 10 repetitions with 1 min rest between sets) and one-repetition maximum (1RM) tests to avoid potential learning effects. Thereafter, all volunteers were randomized (via a computer-generated sequence in the website: https://www.random.org) and counterbalanced to receive one of two treatments (active or placebo laser) on two occasions (T1 and T2) separated by a 7-day washout period. In this design, participants served as their own controls. During T1 and T2, participants received their respective treatments (active or placebo laser) on the biceps brachii muscle of both arms at 20 min before the repetitions-to-failure test (six sets at 60% of 1RM until failure, with 60-s rest between sets) for preacher curl exercise (elbow flexion) with barbell. RPE via the OMNI-Resistance Exercise Scale (OMNI-RES) (Robertson et al., 2003) was recorded immediately before the next set began (after the 60-s rest). Finally, the visual analog scale (VAS) was recorded at 0 h (basal–pretest) and 30 min, 24 h, 48 h, and 72 h after the repetitions-to-failure test to analyze the muscle soreness over time of recovery.

### Participants

Fifteen previously untrained young adult women were recruited from a university population, and 13 of them completed the study (age, 20.1 ± 2.9 years; height, 160.8 ± 5.0 cm; weight, 56.9 ± 7.2 kg; body mass index, 22.0 ± 2.1 kg/m^2^; elbow flexion one-repetition maximum, 6.4 ± 1.4). Two participants withdrew due to factors not related to the study. Sample size calculation for an *F*-test was performed using the software G^∗^Power (Axel Buchner, Version 3.0.1; Düsseldorf, Germany) and was based on previous studies that analyzed the effects of laser PBM on the number of repetitions performed (Leal Junior et al., 2009) and RPE (Toma et al., 2018). Based on a statistical power (1 − β) of 0.95, a moderate effect size (0.65) (Leal Junior et al., 2009; Toma et al., 2018), and an overall level of significance of 0.05, at least 12 participants were required for this study. The inclusion criteria were the following: (i) aged 18–30 years, (ii) classified as eutrophic [i.e., body mass index (BMI) range, 18–25 kg/m^2^], and (iii) classified as low risk for vigorous exercising and testing, according to criteria proposed by the American College of Sports Medicine (Ferguson, 2014). All volunteers were screened with the Physical Activity Readiness Questionnaire (PAR-Q) and were excluded if they (i) were tobacco product users, (ii) used any ergogenic supplement within 6 months prior to the start of the study, (iii) were taking any medication that could affect the ability to perform the physical tests, or (iv) had any physiological (e.g., cardiorespiratory and metabolic diseases) or physical limitation (e.g., orthopedic diseases, muscular injury, or musculoskeletal pain) that could affect the ability to perform the physical test. All women were eumenorrheic with a normal menstrual cycle length of 25–32 days and were in the follicular phase of their menstrual cycle. This study was approved by the Institutional Review Board of the University and conducted in accordance with the latest revision of the Declaration of Helsinki. After a detailed explanation of the procedures, risks, and benefits of this investigation, all participants gave their informed and written consent before starting the study.

### Photobiomodulation Protocol

**FIGURE 2:**
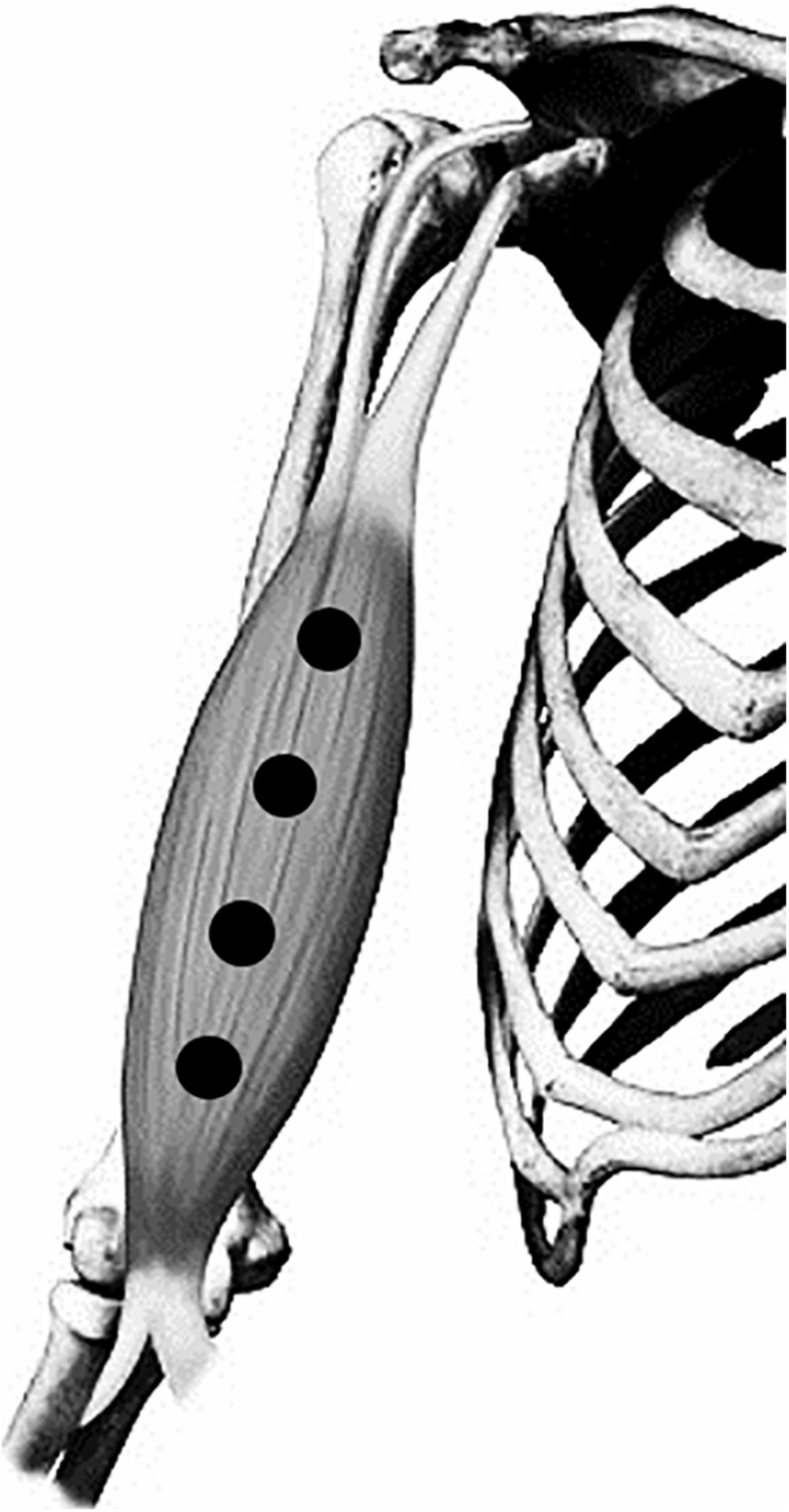
Treatment points (black circles) over biceps brachii muscle.

Participants received placebo or active laser irradiation on the biceps brachii muscle of both arms at 20 min before repetitions-to-failure test during T1 and T2. The irradiation parameters are shown in Table 1. A researcher who was not involved in laser application was responsible for preparing the probe parameters (active or placebo), and an adhesive tape was used to cover the specifications written in the laser probe. Another researcher blinded to the treatment conditions applied the irradiation on four points distributed over the muscle belly (Figure 2; Leal Junior et al., 2009; de Almeida et al., 2012), using an infrared AsGaAl laser (λ, 808 nm) equipment (Therapy XT; DMC^®^ São Carlos, SP, Brazil). Points were placed at 50, 60, 70, and 80% of the total distance between acromion process of the scapula and the antecubital fossa. The laser parameters were based on a previous meta-analysis study that suggested an energy dose range from 20 to 60 J for small muscular groups (Vanin et al., 2018). Participants and therapists were blinded using opaque goggles during the PBM procedures. The goggles also served to protect the eyes against irradiation.

**TABLE 1:** Laser parameters.

Wavelength	808 nm
Frequency	Continuous output
Optical output	100 mW
Irradiance	35.7 W cm^–2^
Energy	7 J each point
Spot size	0.028 cm^2^
Fluency	250 J cm^–2^
Time per point	70 s
Number of points	4
Total energy	28 J
Application mode probe	Stationary in skin contact mode

### One-Repetition Maximum

Elbow flexion 1RM was determined in a bilateral preacher curl exercise with an EZ curl barbell on a seated Scott Bench (Nakagym Equipment, São Paulo, Brazil). The test was preceded by a specific warm-up exercise consisting of two sets of eight and five repetitions at ∼50 and ∼ 60% of an estimated 1RM load, respectively, with a 3-min rest between sets. Thereafter, participants had up to three attempts to achieve the 1RM with a progressive increase in load between each attempt and 5-min rest intervals to allow sufficient recovery. The range of motion of the 1RM test was 10–110° (0° = full elbow extension) and was visually controlled by an experienced rater. Verbal encouragement was provided during each attempt, and the test was standardized and continuously monitored by the same rater to ensure data quality and determine the load within three attempts. The baseline intraclass correlation coefficient (ICC) for test–retest reliability was 0.96 for the 1RM test.

### Repetitions-to-Failure Test

The repetitions-to-failure test consisted of six sets at 60% of 1RM until failure, with 60-s rest intervals between sets, for bilateral preacher curl exercise (elbow flexion) with an EZ curl barbell on a seated Scott Bench (Nakagym Equipment, São Paulo, Brazil). Participants were instructed to keep their hip, knee, and ankle angles flexed at ∼90° and hold the bar with hands supinated and shoulder-width apart. The range of motion was 10–110° (0° = full elbow extension) and was visually controlled by an experienced rater. The repetition cadence was 1/2 s (concentric/eccentric), according to the metronome. For each set, subjects performed as many repetitions as possible until concentric failure (the point in the concentric phase in a set where a full repetition cannot be completed), and the number of maximum repetitions in each set was recorded. This exercise was used to isolate and thus maximize the recruitment of the biceps brachii muscle during the repetitions-to-failure test. We chose a load at 60% of 1RM with 60-s rest between sets to allow participants to complete six sets to failure, in order to investigate the effects of laser PBM on biceps brachii performance to exhaustion. Participants were verbally encouraged by an experienced evaluator (blind to treatment) during each set. The test sessions were carried out between 8:00 and 10:00 a.m., after 12-h overnight fasting. Participants were instructed to wear light clothing, and the water intake was *ad libitum*.

### Rating of Perceived Exertion

Rating of perceived exertion was recorded immediately before the next set began (after the 60-s rest) using the OMNI-RES scale (Robertson et al., 2003). The participants were instructed to report the perceived exertion value by indicating a number on the OMNI-RES scale (0 for “no effort” and 10 for “maximal effort”) that best represented their overall muscular effort (Robertson et al., 2003). The score was the value (0–10) reported on the OMNI-RES scale. Volunteers were familiarized with the OMNI-RES scale before starting the study.

### Muscle Soreness

Muscle soreness was assessed using a 100-mm VAS, with “no soreness” (0 mm) and “severe soreness” (100 mm) as the left and right anchors, respectively. The participants were instructed to palpate the biceps brachii muscle belly of the dominant arm and mark a scale point that best represented their momentary soreness (Rinard et al., 2000; Flores et al., 2011). The score was the distance (in millimeters) from the left side of the scale to the point marked. Palpation was performed in a circular motion and constant pressure in a clockwise direction, with the tips of the index and middle fingers toward the deeper tissues, for approximately 3 s (Lau et al., 2015). Participants practiced palpation prior to starting the study to reproduce the constant pressure within a 5% variation between trials. A previous study described that the VAS ICC is ≥0.97 (Bijur et al., 2001).

### Statistical Analysis

All values are reported as mean and standard deviation (SD). Statistical analyses were performed using a commercially available software package (SPSS Statistics for Windows version 20.0, IBM^®^, Chicago, IL, United States). Normality of data was checked by the Shapiro–Wilk test. Independent variables included the experimental conditions (i.e., placebo and active laser). Dependent variables were consisted of repetitions to failure, RPE, and muscle soreness. Repeated-measures analysis of variance (ANOVA) was used to determine differences between placebo and active laser conditions for all dependent variables. Bonferroni *post-hoc* correction was used to identify the differences confirmed with ANOVA. The Greenhouse–Geisser method was used to correct the violation of sphericity. The significance level was set at *p* ≤ 0.05. Cohen’s *d* (ES) was also calculated to quantify the magnitude of difference between conditions, considering an ES < 0.19 as trivial, 0.20–0.49 as small, 0.50–0.79 as moderate, and ≥0.80 as large (Cohen, 1992).

## Results

All participants were classified as eutrophic (i.e., BMI range, 18–25 kg/m^2^), indicating that body composition had no effect on global results. The sequence of treatments was randomized and counterbalanced between visits (T1, active; T2, placebo vs. T1, placebo; T2, active) in order to ensure there was no order effect associated with treatments (placebo or laser). No volunteer reported adverse effects of laser during the study.

### Repetitions to Failure

**FIGURE 3:**
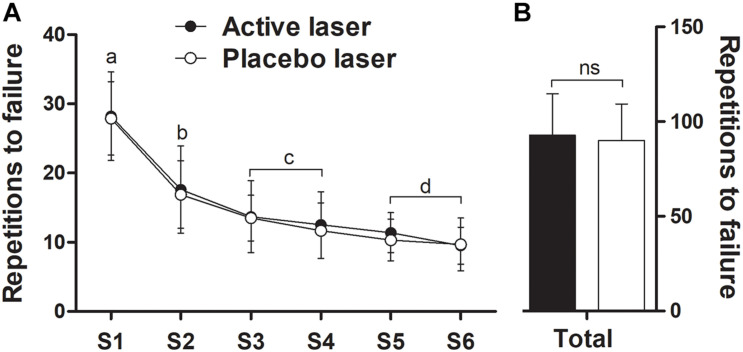
Number of repetitions to failure over six sets (S1–S6) **(A)** and corresponding total repetitions (sum of all sets) **(B)** during elbow flexion exercise in the active and placebo laser conditions (*N* = 13). Data are means ± SD. There were no significant (*p* > 0.05) differences over time between conditions. Different letters indicate significant (*p* < 0.05) difference between time points for both groups.

A significant main effect of time (*p* < 0.05) indicated a reduction in the number of repetitions performed among the sets, with no significant differences (treatment × time, *p* > 0.05) between placebo and active laser conditions (Figure 3A). The total number of repetitions (placebo, 90 ± 19 vs. active, 93 ± 22) over the six sets (Figure 3B) and the corresponding area under curve (AUC) (placebo, 4,276 ± 948 vs. active, 4,442 ± 1,146) were similar (treatment × time, *p* > 0.05) between placebo and active laser conditions. A trivial ES (0.14) was observed between conditions for the total number of repetitions, indicating no beneficial effect in favor of the active laser.

### Rating of Perceived Exertion

**FIGURE 4:**
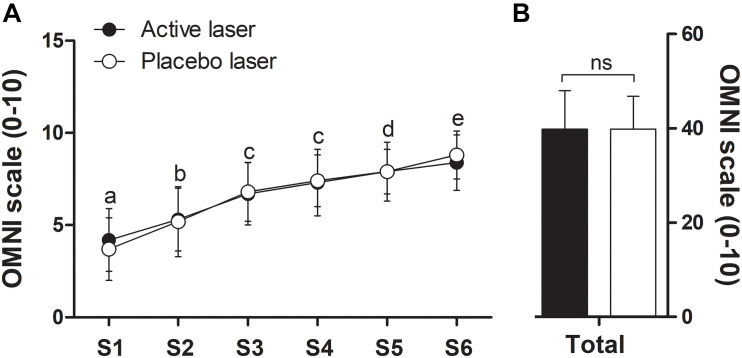
Rating of perceived exertion (RPE) immediately before the next set began (after the 60-s rest) **(A)** and corresponding total RPE (sum of all sets, S1–S6) **(B)** of the repetitions-to-failure test in the active and placebo laser conditions (*N* = 13). Data are means ± SD. There were no significant (*p* > 0.05) differences over time between conditions. Different letters indicate significant (*p* < 0.05) difference between time points for both groups.

There was a significant (time, *p* < 0.05) increase in RPE immediately before the next set began (after 60-s rest interval) (Figure 4A), with no significant differences (treatment × time, *p* > 0.05) between placebo and active laser conditions. Total RPE (sum of all sets) immediately before the next set began (placebo, 39.8 ± 7.0 vs. active, 39.8 ± 8.2) (Figure 4B) and the corresponding AUC (placebo, 2,017 ± 376 vs. active, 2,010 ± 430) were similar (*p* > 0.05) between placebo and active laser conditions. A negligible ES (0.00) was observed between conditions for the total RPE (sum of all sets), indicating no beneficial effect in favor of the active laser.

### Muscle Soreness

**FIGURE 5:**
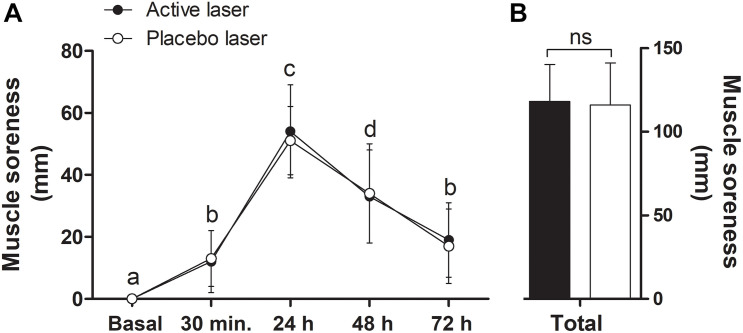
Delayed onset muscle soreness (DOMS) during the recovery days (basal to 72 h postexercise) **(A)** and corresponding total DOMS (sum of all times) **(B)** in the active and placebo laser conditions (*N* = 13). Data are means ± SD. There were no significant (*p* > 0.05) differences over time between conditions. Different letters indicate significant (*p* < 0.05) difference between time points for both groups.

There was no significant condition × time interaction (*p* > 0.05), but a significant main effect of time (*p* < 0.05) showed an increase in DOMS from basal to 30 min and 24 h postexercise and progressively returned to baseline at 48 and 72 h post repetitions-to-failure test in both placebo and active laser conditions (time, *p* < 0.05) (Figure 5A). Total DOMS (Figure 5B) and the corresponding AUC (placebo, 2,850 ± 601 vs. active, 2,957 ± 554) were similar (*p* > 0.05) between conditions. A trivial ES (0.19) between treatments indicated no beneficial effect in favor of the active laser.

## Discussion

The purpose of this investigation was to evaluate the effects of laser PBM at 808 nm on biceps brachii performance to exhaustion and DOMS in untrained young adult women. Considering previous positive findings in men, we hypothesized that laser PBM would increase muscle performance to exhaustion (i.e., increased number of maximum repetitions and reduced RPE) and attenuate DOMS when compared to a placebo laser. Contrary to our hypothesis, our findings showed no significant difference in the number of repetitions to failure, RPE, and DOMS between placebo and active laser conditions, indicating that the effects of laser PBM on biceps brachii performance to exhaustion or attenuation of exercise-induced DOMS may be negligible in young adult women.

Previous studies have shown different endurance capacity (e.g., number of repetitions performed at a given percentage of 1RM) in lower- and upper-body muscles (Hoeger et al., 1987, 1990; Shimano et al., 2006), probably due to the amount of muscle mass (Shimano et al., 2006) and joints (e.g., single- or multijoints exercises) involved as well as the composition of fiber types. These physiological (i.e., endurance capacity), morphological (i.e., amount of muscle mass, and fiber-type composition), and mechanical (i.e., amount of joints) differences between lower- and upper-body limbs may potentially influence the effects of laser PBM on muscle tissue. Furthermore, the larger size of the lower limb muscles (e.g., quadriceps) compared to the upper body muscles (e.g., biceps) allows irradiation to be applied to a greater number of points, resulting in greater total energy delivered to the muscle and consequently stimulus for performance and adaptations. All these points indicate that a non-dissimilar analysis between different muscles (e.g., lower and upper limbs) may complicate the interpreting and understanding of the effects of PBM on the performance of specific muscles. Therefore, to avoid these confounding factors, we conducted a comparative analysis only with performance data from studies that analyzed the effects of laser PBM on biceps brachii muscle.

Similar to our study, Higashi et al. (2013) found no positive effect of laser PBM on markers of endurance performance (i.e., number of repetitions performed, EMG fatigue, and blood lactate levels) on the biceps brachii in untrained healthy women. It is worth mentioning that the doses applied in our studies (28 and 56 J) are within the dose range (20–60 J) recommended for small muscular groups (Vanin et al., 2018), indicating that this “therapeutic window” may not be effective for women. Consistent with this premise, previous studies involving only men showed improvement in muscle performance (Leal Junior et al., 2008, 2009, 2010; de Almeida et al., 2012), but no positive effect was found on muscle performance or DOMS when only women (Higashi et al., 2013) or a mix of men and women (Craig et al., 1999) were recruited. Albeit the exact mechanisms that explain the influence of sex on PBM-induced muscle adaptations remain unknown, a possible explanation may be the difference in the endurance ability of biceps brachii between men and women. Previous studies reported that women have greater resistance to fatigue than men in multiple sets of repetitions to failure (Salvador et al., 2005) and maximum voluntary isometric contraction (MVIC) to failure (Avin et al., 2010) involving elbow flexion exercise. This greater fatigue resistance could mask a small effect magnitude of the laser PBM on biceps brachii muscle performance to exhaustion in women, suggesting that the effects of PBM may be influenced by sex-related muscle differences. Several factors have been postulated to explain the differences in fatigue resistance between sex, including substrate utilization, muscle morphology, and neuromuscular activation (Hicks et al., 2001), but it remains unknown how these factors may affect muscle adaptations induced by laser PBM. In addition, we cannot rule out the possibility that reproductive hormones (e.g., menstrual cycle) may alter the endurance performance of women (Julian et al., 2017) and, consequently, influence the ergogenic effects of PBM. Future studies are needed to elucidate these points.

In this study, we also show the first data evaluating the effects of laser PBM on RPE after exercise to failure for biceps brachii muscle in young adult women. We found no significant difference in RPE at 60 s after each set between active and placebo laser conditions. Consistent with our findings, Felismino et al. (2014) using a laser at 808 nm applied on biceps brachii reported no beneficial effect on RPE after biceps curl exercise, consisting of 10 sets of 10 repetitions at 50% of 1RM in young adult men. This lack of effect of laser PBM on RPE corroborates the non-significant findings of biceps brachii performance found in our study and others (Higashi et al., 2013) involving only young adult women. However, Toma et al. (2018) reported a lower RPE after an isokinetic fatigue test and greater muscle performance in the laser group, compared to control, in young adult women. This lack of effects of laser PBM on RPE and muscle performance in our study and others involving biceps brachii muscle (Higashi et al., 2013) in contrast to the positive effects observed on the quadriceps muscle (Toma et al., 2018) raises the possibility that laser PBM may have different effects on small and large muscles. However, this supposition is not supported by recent studies that have found no positive effect on physical performance and markers of muscle recovery and damage following the PBM irradiation by light-emitting diodes (LEDs) or laser to the larger muscles or the whole body in young adult men (Zagatto et al., 2016, 2020; Malta et al., 2018; Peserico et al., 2019; Dutra et al., 2020; Ghigiarelli et al., 2020). Therefore, in light of our findings and that of others, its seems that the lack of positive effect of PBM is more associated with the inherent ineffectiveness of the therapy itself than with other factors, such as the type of irradiation (laser or LED), sex, and irradiated muscle.

Finally, we have found no significant difference in the DOMS up to 72 h after repetitions-to-failure exercise between active and placebo laser conditions. To our knowledge, no previous study has investigated the effects of laser PBM on DOMS after exercise to failure in a sample including only women. Our results are similar to previous studies involving only men (Craig et al., 1996; Baroni et al., 2010b; Cieśliński et al., 2018) or a mix of men and women (Craig et al., 1999; Kakihata et al., 2015) where no positive effect was found on DOMS following different DOMS induction protocols, including (i) a single session of neuromuscular electrical stimulation of the quadriceps femoris muscle (Cieśliński et al., 2018), (ii) five sets of 15 repetitions for knee extensors exercise at velocity = 60°seg^–1^ (Baroni et al., 2010a), (iii) six vertical jumps lasting 60 s (Kakihata et al., 2015), (iv) repeated eccentric contractions of the elbow flexors until exhaustion (Craig et al., 1996, 1999), and (v) three 3-s isometric contractions of the elbow flexors (Kobordo, 2015). In addition, our findings support the evidence from a recent review (Nampo et al., 2016) in which authors reported limited effectiveness of laser PBM on DOMS. Therefore, further studies are needed to determine the effectiveness of the laser PBM on DOMS, especially in women.

This study has some limitations that should be mentioned. First, we have analyzed the acute effects of laser PBM on muscle performance and DOMS, so we cannot rule out the possibility of a positive effect if the laser is applied chronically or in combination with a resistance exercise program. Second, we have only analyzed the performance of the upper-extremity muscle (i.e., the biceps brachii), so we cannot rule out the possibility of a beneficial effect if the laser is applied to lower-extremity muscles (e.g., quadriceps). Third, we did not use physiological fatigue (e.g., lactate and EMG signal) and recovery (e.g., blood CK levels) markers to corroborate functional performance data, but previous studies have shown that laser PBM does not improve the lactate levels and EMG fatigue index in young women (Higashi et al., 2013). Finally, we did not include the analysis of muscle function (e.g., force production) to corroborate the DOMS data during the recovery from intense exercise. Future studies are required to address these issues.

In conclusion, our results indicate that acute laser PBM (808 nm) at a dose of 28 J does not improve biceps brachii performance to exhaustion, RPE, and DOMS in untrained young women. Therefore, it seems premature to consider acute laser PBM therapy as a potential strategy to improve muscular endurance and recovery of DOMS in this population. Further studies are warranted to confirm whether other laser PBM settings (e.g., wavelength, energy density, and total dose) may provide some benefit in this population or in others (e.g., men and elderly) in different muscles (e.g., lower body vs. upper body) under different clinical (e.g., healthy or not) and training conditions (e.g., trained vs. untrained).

## Data Availability Statement

The raw data supporting the conclusions of this article will be made available by the authors, without undue reservation.

## Ethics Statement

The studies involving human participants were reviewed and approved by the Research Ethics Board of the University of Northern Paraná. The patients/participants provided their written informed consent to participate in this study.

## Author Contributions

RA, JM, JJ, and JB contributed to data collection and analysis. RS, FP, JN, and AR participated in the design of the study, contributed to data analysis, and interpretation of results. AA participated in the conception and initial design of the project, and contributed to data analysis and interpretation of results. All authors contributed to the manuscript writing, read and approved the final version of the manuscript, and agreed with the order of presentation of the authors.

## Conflict of Interest

The authors declare that the research was conducted in the absence of any commercial or financial relationships that could be construed as a potential conflict of interest.
